# Global Processing Makes People Happier Than Local Processing

**DOI:** 10.3389/fpsyg.2019.00670

**Published:** 2019-03-26

**Authors:** Li-Jun Ji, Suhui Yap, Michael W. Best, Kayla McGeorge

**Affiliations:** Department of Psychology, Queen’s University, Kingston, ON, Canada

**Keywords:** global processing, local processing, happiness, mood, perceptual cognitive processing

## Abstract

Past research demonstrates that mood can influence level of perceptual processing (global vs. local). The present research shows that level of perceptual processing can influence mood as well. In four studies, we manipulated people’s level of perceptual processing using a Navon letter task (Study 1), landscape scenery (Study 2), and Google Maps Street View images (Studies 3 and 4). Results from these studies and a meta-analysis support the conclusion that global processing results in higher happiness than local processing. In conjunction with previous findings that mood affects level of cognitive processing, these results suggest that the link between level of processing and mood may be reciprocal and bidirectional.

## Introduction

If you broaden your perspective, and look at the situation from a global perspective, then you might see some basis for hope. – Dalai Lama.

In *The Art of Happiness in a Troubled World* (Lama and Cutler, 2009), Dalai Lama discussed how happiness can be achieved by adopting a wider or global perspective, which he believes is extremely helpful for maintaining hope and coping with troubled situations. People often attend to and process information around them from two different perspectives – global vs. local processing styles (Navon, 1977). Global processing style refers to attending to the Gestalt of a stimulus, or processing information in a more general and big-picture way, whereas local processing style refers to attending to the specific details of a stimulus or processing information in a narrower and a more detail-oriented way (Navon, 1977; Kimchi, 1992). The present research empirically examines how global vs. local perceptual processing can affect happiness.

A plethora of studies has documented various triggers of processing styles, and one of the factors determining whether someone processes information at a local or global level is a person’s mood (e.g., Kimchi, 1992; Derryberry and Tucker, 1994; Basso et al., 1996). Specifically, research shows that positive moods are more likely to elicit a global level of processing, whereas negative moods are more likely to elicit a local level of processing (Bless et al., 1996; Clore et al., 2001; Gasper and Clore, 2002; Gasper, 2004; Fredrickson and Branigan, 2005). For instance, participants who wrote about a happy and positive life event were more likely to match the figures in the Kimchi–Palmer-task based on their global features (instead of local features) than participants who wrote about a sad and negative life event (Gasper and Clore, 2002). Fredrickson and Branigan (2005) also demonstrated that, compared to participants in a neutral mood state, participants in a positive mood were more likely to broaden their scope of attention and matched figures based on their global configuration, whereas participants in a negative mood were more likely to narrow their scope of attention and matched figures based on their local elements.

One explanation for this phenomenon lies in the affect-as-information hypothesis (Schwarz and Clore, 1983; Clore, 1992). According to this hypothesis, mood serves as information to help people understand their current situation. Under this model, positive mood signals a benign situation and results in more global and heuristic processing. On the other hand, negative mood signals that the situation is problematic and that detailed and systematic processing is necessary. If mood signals whether a situation is problematic or not, then global and local processing styles – usually in response to a benign or problematic situation, respectively, may help to reinforce the desirableness (or favorableness) of the situation, further resulting in more positive mood associated with global than with local processing styles.

Another explanation for the effect of mood on processing styles is based on mood maintenance motivation, which suggests that motivation may mediate the relationship between mood and level of processing (Isen, 1987; Wegener et al., 1995; Clore et al., 2001). Effortful, detail-oriented, local processing may draw attention to details that could potentially result in a reduction of mood (Clore et al., 2001). Thus, when in a positive mood, people may be less motivated to engage in systematic local processing and more motivated to engage in heuristic processing that relies on global scripts and schemes in order to avoid potential mood reduction (Bless and Fiedler, 1995; Bless et al., 1996; Gasper and Clore, 2002). This explanation suggests that global processing may help maintain a positive mood, whereas local processing may dampen it.

Past research has established a correlation between processing level and mood. For example, Basso et al. (1996) found that global processing was positively correlated with individuals’ trait happiness and negatively correlated with individuals’ trait depression. The tendency to focus on details and individual parts of a situation, instead of focusing on the big picture, has also been associated with depressive symptomatology (Weintraub et al., 1974; Msetfi et al., 2005). Furthermore, correlational research on clinical and sub-clinical depression suggest that information processing styles may affect the development and maintenance of negative mood in depressive individuals (Erickson et al., 2005; Mathews and MacLeod, 2005; Koster et al., 2011). Collectively, these findings provide evidence that processing styles are associated with mood. The nature of this association, especially the effect of processing level on individuals’ mood state, however, has received relatively little attention in research.

A few recent studies have suggested the possibility of a causal relationship whereby broadened and global processing can promote positive mood (Bar, 2009; Srinivasan and Hanif, 2010; Chermahini and Hommel, 2012; Brunye et al., 2013; Updegraff and Suh, 2007). For example, Srinivasan and Hanif (2010) found that global processing facilitated the identification of happy faces, whereas local processing facilitated the identification of sad faces. This implies that global level of processing may have potentially directed one’s attention to positive mood stimuli (e.g., happy faces), which can be helpful in maintaining or promoting one’s happy mood. Likewise, other researchers have shown that broadened associative thinking or divergent thinking led to a more positive mood, whereas narrow associative thinking or convergent thinking led to a more negative mood (Bar, 2009; Chermahini and Hommel, 2012). Furthermore, Brunye et al. (2013) demonstrated that individuals engaging in broad associative processing were more likely to report a slight boost in positive affect and a significant decrease in negative affect, compared to individuals engaging in narrow and constrained associative processing. In addition, Updegraff and Suh (2007) showed that people who thought about themselves in more abstract terms reported greater increase in life satisfaction than those who thought about themselves in more concrete terms. Together, these findings suggest that individuals’ processing styles, conceptual or perceptual, can potentially influence their mood state.

To be sure, there are distinctions between conceptual and perceptual processing: perceptual processing refers to organizing sensory inputs into meaningful patterns (such as making sense of visual stimuli), whereas conceptual processing refers to the activation of concepts and semantic associations in memory (Förster and Dannenberg, 2010). Despite their differences, perceptual processing styles are associated with conceptual processing styles (Finke, 1985; Martindale, 1995; Förster and Dannenberg, 2010). Van Dantzig et al. (2008) showed that pure perceptual processing of stimuli (perceiving stimuli without any semantic meaning) affected the activation of the conceptual knowledge, indicating that perceptual and conceptual processing styles are “partially based on the same systems” (p. 585). Along the same line, researchers also demonstrated that experimentally broadening individuals’ perceptual attention widens their conceptual attention, thereby activating more abstract and remote associations in creativity tasks (Friedman and Förster, 2001; Friedman et al., 2003). As such, given the possibility of a causal relationship between conceptual processing and positive mood (Bar, 2009; Chermahini and Hommel, 2012; Brunye et al., 2013), and the connection between conceptual and perceptual processing styles, one may expect individuals’ perceptual processing to influence their mood state in a similar way as conceptual processing.

Neurological evidence also suggests connections between affective and cognitive processes, through their overlapping neuropsychological networks within the hippocampus and parahippocampal cortex in the medial temporal lobes (MTL) and medial prefrontal cortex (MPFC) (Ashby et al., 1999; Bar, 2009). Within these brain regions, the contextual associations neural network, which is implicated in broad vs. narrow associative processing, showed remarkable overlap with the cortical network, which is associated with depressive symptoms observed in mood disorders (Raichle et al., 2001; Bar et al., 2007). Narrow associative processing and the exacerbation of depressive symptoms are found to be associated with decreased activities in the MTL and MPFC, whereas broadened associative processing and the alleviation of depressive symptoms are associated with the restoration of activities in the MTL and MPFC to their default levels (Joormann, 2004; Mayberg et al., 2005). Furthermore, positive affect is also associated with the release of dopamine in the brain, which is associated with the increased release of acetylcholine that plays an important role in the normal functioning of the hippocampus in the MTL (Ashby et al., 1999). Given that both cognitive and affective processes share a common brain network, and are associated with dopamine release in the brain, it is possible that broadened global cognitive process can promote positive mood via their overlapping neuropsychological pathways. Nevertheless, evidence involving experimental manipulation of processing levels is needed to provide direct evidence for the causal effect of processing levels on mood.

Unlike previous studies that manipulated individuals’ conceptual processing (e.g., Chermahini and Hommel, 2012; Brunye et al., 2013), the current research manipulated individuals’ global and local perceptual processing, and examined its effect on mood. In four studies, we manipulated global and local perceptual processing styles by asking people to focus on the global or local structure of composite letters in a Navon letter task (Study 1), to focus on the global or local perspective of landscape pictures (Study 2), and Google Maps Street View pictures (Studies 3 and 4). Based on previous research demonstrating a link between global processing and positive mood, and between local processing and negative mood, we hypothesized that global processing would make people happier than local processing.

## Study 1

In Study 1, all participants had negative mood induced before engaging in either global or local processing with a Navon letter task. We examined whether global processing would elevate one’s self-reported mood more than local processing.

### Participants

Eighty Euro-Canadian university students (62 women and 18 men, *M*_Age_ = 18.2, *SD*_Age_ = 1.0) completed the study for course credit^1^.

### Materials and Procedure

The study was conducted in a quiet lab room. The participant was seated before a computer, 60 cm away from the screen. The participant first viewed a sad movie clip from the movie *The Champ*, previously found to elicit sad mood (Gross and Levenson, 1995). We induced an initial negative mood state in participants due to a concern for a possible ceiling effect: Euro-Canadians are one of the happiest people in the world according to Gallup world polls from 2005 to 2014, and thus might have quite a positive mood as their baseline state^2^.

After viewing the movie clip, participants completed the first mood measure by answering how happy they felt at the moment on a 10-point scale (1 = *least happy*, 10 = *most happy*). Afterward, participants completed a letter-identification task, adapted from the Navon Letters task, on the computer (Navon, 1977; Förster et al., 2008). This task was designed to manipulate individuals’ global and local perceptual processing styles. Each stimulus used for this letter-identification task was an image of a composite letter – a large letter (5 cm × 5 cm) made up of mismatching smaller letters (1 cm × 1 cm). For example, a large letter *I* was made up of smaller letter *H*s. In this case, the letter *I* would be considered as the global target, and the *H*s would be considered as the local elements. The composite letters were created using nine different alphabets (F, H, I, K, N, T, X, Y, Z). Participants were randomly assigned to identify the global targets (global condition), or the local targets (local condition). During each trial, participants were presented with a fixation cross (+) in the center of the screen for 1.5 s prior to the onset of the stimulus. A composite letter was then randomly presented for 0.5 s. Depending on the condition they were assigned to, participants selected the letter that either matched the global or local target from among the nine alphabets presented to them. Afterward, they received feedback as to whether their choice was correct or incorrect. This feedback was to make sure that participants followed the instructions of the task so that we could effectively induce the respective cognitive processing styles. The feedback remained on the screen for 1.5 s before the next trial began. There were 30 trials in total (including one practice trial).

In order to administer the same mood measure a second time without arousing any suspicions about the purpose of our study, we pre-programmed a bogus error message. After participants completed the letter-identification task, a pre-programmed error message appeared on the computer screen. Participants were led to believe that the program had crashed unexpectedly and that all of their data were lost. Participants were then asked to complete another quick experiment instead, in which they reported their current mood again by completing the same mood measure they had done earlier, embedded in a few other measures unrelated to the present study.

Lastly, participants reported their demographic information such as age, ethnicity, and year in university, and then watched a happy clip from the movie *When Harry met Sally.* This was to ensure that all participants were in a positive mood before leaving the lab. Once the experiment was completed, participants were fully debriefed about the deception regarding the “error” message.

### Results

A one-way analysis of covariance (ANCOVA), with pre-manipulation happy mood as a covariate, was conducted to examine the effects of processing style on post-manipulation happy mood. Controlling for pre-manipulation happy mood, the ANCOVA revealed a significant main effect of condition, *F*(1, 71) = 4.23, *p* = 0.044, partial η^2^ = 0.06^3^. Participants who processed the stimuli globally (adjusted marginal *M* = 6.01, *SE* = 0.14) reported a happier mood than those who processed stimuli locally (adjusted marginal *M* = 5.58, *SE* = 0.15), *d* = 0.495, 95% CI [0.03, 0.96]^4^. Alternatively, a 2 (Condition: global vs. local) × 2 (Time: pre- vs. post-processing task) repeated ANOVA revealed a marginally significant interaction effect between condition and time, *F*(1, 72) = 3.07, *p* = 0.084, partial η^2^ = 0.04. Follow up simple effects showed that local processing led to a decrease in participants’ happiness (*M*_pre_ = 5.53, *SE* = 0.27; *M*_post_ = 5.18, *SE* = 0.28), *F*(1, 72) = 5.67, *p* = 0.020, 95% CI [−0.65, −0.06], but global processing did not change happiness (*M*_pre_ = 6.35, *SE* = 0.25; *M*_post_ = 6.35, *SE* = 0.26), *F*(1, 72) = 0.00, *p* > 0.25.

## Study 2

One limitation of Study 1 is that the bogus error message about the program crashing might have affected participants’ post-manipulation mood in some ways, and that could potentially complicate the experimental procedure and introduce unnecessary noise into the data. Therefore, Study 2 dropped the bogus error message and used more straightforward instructions when re-administering the measure of mood. In addition, to increase generalizability, Study 2 manipulated participants’ perceptual processing styles by getting them to focus on the global vs. local aspect of landscape images instead of Navon letters.

### Participants

Sixty-two Euro-Canadian university students (41 women and 21 men; *M*_Age_ = 18.66, *SD*_Age_ = 0.83) participated in the study for course credit in their introductory psychology course.

### Materials

**FIGURE 1:**
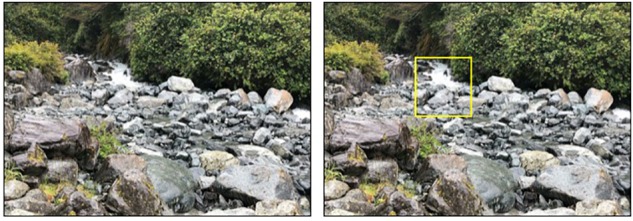
A sample of global (left) and local (right) landscape view stimuli (Study 2).

Adapting from Friedman et al. (2003) map task, we used images of neutral landscape to induce global and local processing. For the global stimulus, the entire image of the landscape (16 cm × 12 cm) was presented as the global target (see Figure 1). For the local stimuli, the same landscape images were used except that a random local target area of each image (3 cm × 3.5 cm) was delineated by a yellow outline (see Figure 1). Hence, participants in both global and local conditions were presented with the same landscape images. The location of the delineated target area varied slightly for each image (i.e., from the center of the page, center-right, center-left, a little above center and a little below center, etc.) so that participants would actively scrutinize the contents of the various local target area, instead of passively focusing their attention on the same location of the screen.

Eleven images of landscapes from Google search were selected based on a pretest, in which a separate group of 76 participants rated the pleasantness of the global vs. local versions of the images on a scale from 1 (*not pleasant at all*) to 7 (*extremely pleasant*). The local areas within the yellow boxes (*M* = 4.68, *SD* = 1.02) were rated as similarly pleasant as their corresponding global images (*M* = 4.89, *SD* = 0.87), *F*(1, 74) = 0.91, *p* = 0.34.

### Procedure

As in Study 1, participants were seated at a fixed distance in front of the computer in a quiet room. They viewed the sad movie clip as in Study 1 and completed the first mood measure by answering how happy they felt at the moment on a 10-point scale (1 = *least happy*, 10 = *most happy*)^5^. Participants were then randomly assigned to either the global or local processing condition. To induce global processing, they were given 5 s to look at the entire landscape image as a whole, and were then instructed to describe in a few sentences what they saw. To manipulate local processing, they were instructed to only focus on the area delimited by the yellow box (and ignore the rest of the image) for 5 s, and then describe in a few sentences what they saw in the yellow box of the image. As an excuse to measure their mood twice, we told participants that they would report their mood again on a different colored background for us to examine the possible effect of background color on mood. This new instruction was not only more straightforward but also implied that it would be reasonable for participants to report different ratings on the same mood measure. Thus, the happiness mood item was presented on a slightly different neutral colored background (two different shades of light gray) before and after the experimental manipulation. These colored backgrounds were counterbalanced across all participants.

Finally, participants reported their demographic information and watched the happy movie clip to ensure that they were in a positive mood before they were fully debriefed.

### Results

As in study 1, a one-way ANCOVA, with pre-manipulation happy mood as a covariate, revealed a significant main effect of condition on happy mood, *F*(1, 55) = 5.40, *p* = 0.024, partial η^2^ = 0.09^6^. Supporting our prediction, participants who processed the stimuli globally (adjusted marginal *M* = 6.74, *SE* = 0.20) reported a happier mood than those who processed stimuli locally (adjusted marginal *M* = 6.12, *SE* = 0.18), *d* = 0.62, 95% CI [0.09, 1.15]. Alternatively, a 2 (Condition: global vs. local) × 2 (Time: pre- vs. post-processing task) repeated ANOVA revealed a significant interaction effect between condition and time, *F*(1, 56) = 4.61, *p* = 0.036, partial η^2^ = 0.08. Follow up simple effects analyses revealed that participants’ happiness mood increased marginally after global processing (*M*_pre_ = 6.46, *SE* = 0.28; *M*_post_ = 6.81, *SE* = 0.30), *F*(1, 56) = 2.82, *p* = 0.099. Local processing lowered participants’ happiness mood, although this effect was not statistically significant, (*M*_pre_ = 6.31, *SE* = 0.26; *M*_post_ = 6.06, *SE* = 0.27), *F*(1, 56) = 1.81, *p* = 0.184.

## Study 3

Study 3 aimed to replicate the results from Studies 1 and 2 using a larger sample size and a different manipulation of perceptual processing to examine the generalizability of the findings.

### Participants

One hundred and thirty-four Euro-Canadian university students (111 women and 23 men, *M*_Age_ = 18.50, *SD*_Age_ = 0.94) participated in the study for course credit in their introductory psychology course.

### Materials

All materials (e.g., movie clips and mood measures) used in Study 3 were similar to those used in Study 2, except that we used images of Google Maps Street Views (16 cm × 12 cm) to manipulate global and local perceptual processing. For the global stimuli, the entire image of the Google Street Views was presented as the global target. For the local stimuli, the same Google images were used except that a random local target area of each image (3 cm × 3.5 cm) was delineated by a red outline. Hence, participants in both global and local conditions saw the same Google Street View images. Similar to Study 2, the location of the delineated area varied slightly for each image so that participants would actively scrutinize the contents of the local target area.

Images of Google Maps Street Views were selected from cities in the United States, and we made sure these street views were not from any famous streets. 14 images were selected based on a pretest with a separate group of participants (*N* = 78), in which participants saw the whole images (for the global stimuli) or the images within the red box only (for the local stimuli) and rated how pleasant each image was (1 = *not pleasant at all*; 7 = *extremely pleasant*). The selected local (*M* = 3.39, *SD* = 1.38) and global (*M* = 3.51, *SD* = 1.31) images did not differ in pleasantness (*F*s < 1, *ps* > 0.25).

### Procedure

In a quiet room, the experiment proceeded in the same manner as in Studies 1 and 2. Participants watched the sad movie clip from *The Champ*, rated their current happiness mood^7^, and then were randomly assigned to either the global or local processing condition. Each picture was presented to participants for 5 s, in a random order. Participants in the global condition were asked to look at “the entire picture as a whole,” whereas participants in the local condition were asked to focus only on the “area of the image that is delimited by a red box,” and to ignore the rest of the picture. To make sure participants were paying attention to the stimuli while preventing fatigue and boredom at the same time, we asked participants to describe the image in the picture they last saw at random intervals. Participants in both conditions viewed and described five out of the 14 pictures.

Participants were then asked to rate their current mood again. As in Study 2, we told participants that the background color may influence participants’ responses and that we wanted them to rate their happiness on a different background color. In reality, there was no change in the neutral light gray background color. Afterward, participants answered demographic questions, and watched a happy movie clip before being debriefed.

### Results

Similarly, a one-way ANCOVA, with pre-manipulation happy mood as a covariate, was conducted to examine the effects of processing style on post-manipulation happy mood. A significant main effect of condition, *F*(1, 130) = 5.32, *p* = 0.023, partial η^2^ = 0.04, was found^8^. As predicted, participants in the global processing condition (adjusted marginal *M* = 6.04, *SE* = 0.13) reported a happier mood than those in the local processing condition (adjusted marginal *M* = 5.61, *SE* = 0.14), *d* = 0.398, 95% CI [0.05, 0.74]. Alternatively, a 2 (Condition: global vs. local) × 2 (Time: pre- vs. post-processing task) repeated ANOVA revealed a significant interaction effect of condition by time, *F*(1, 131) = 4.77, *p* = 0.031, partial η^2^ = 0.04. Participants’ happiness mood decreased after local processing (*M*_pre_ = 6.00, *SE* = 0.21; *M*_post_ = 5.64, *SE* = 0.19), *F*(1, 131) = 5.59, *p* = 0.020, 95% CI [−0.67, −0.06], while there was no significant increase in mood after global processing (*M*_pre_ = 5.91, *SE* = 0.20; *M*_post_ = 6.01, *SE* = 0.18), *F*(1, 131) = 0.47, *p* > 0.25.

## Study 4

Consistent with our prediction, Studies 1–3 showed that global processing made people happier than local processing, after controlling for pre-manipulation happiness. One common procedure in these studies was to let participants watch a sad video to put them in an initial unhappy mood. However, doing so could potentially limit the ecological validity of the study, and introduce unnecessary confounds (e.g., effects of the movie overshadowing effects of manipulation). Therefore, in Study 4, we aimed to replicate early studies without inducing a negative mood state in participants prior to the experimental manipulation. It would be important to determine whether the effect of processing on happiness holds even in the absence of an initial sadness induction. In addition, we used multiple indices to measure mood, instead of happiness only, to provide a more comprehensive evaluation of the effect of different processing styles on one’s positive mood in general. Furthermore, we included a control condition, following a similar procedure by Förster et al. (2008), to explore whether global processing enhances mood and whether local processing reduces it.

### Participants

Study 4 involved 70 Euro-Canadian university students (53 women and 17 men, *M*_Age_ = 18.19, *SD*_Age_ = 1.3). They received course credit for their participation.

### Materials and Procedure

Study 4 used the same experimental manipulation as in Study 3 – Google Maps Street View stimuli. First, participants reported their baseline mood by stating how “positive” and how “negative”, respectively, they were feeling at the moment on a scale from 0 (*Not at all*) to 6 (*Very much*). Next, participants were randomly assigned to three conditions (i.e., global, local and control) and viewed the Google Maps images. As in Study 3, participants in the global condition were asked to focus on “the entire picture as a whole,” whereas participants in the local condition were asked to focus only on the “area of the image that is delimited by a red box”, and to ignore the rest of the picture. In the control condition, half of the stimuli used were global stimuli and the other half were local stimuli^9^. More specifically, participants in the control condition were asked to briefly “describe the entire picture you just saw” if the last image they saw was a global stimulus, and to briefly “describe the area in the red box of the picture you just saw” if the last image they saw was a local stimuli. After the Google Maps task, participants reported their positive mood by indicating how *happy, elated, joyful, pleasant, and pleased* they were feeling at the moment, on a scale from 0 (*Not at all*) to 6 (*Very Much*). Also, for exploratory purposes, we asked participants to report their negative mood by indicating how *sad, blue, depressed, gloomy, and unpleasant* they were feeling at the moment on the same 7-point scale. These mood items were adapted from Gasper and Clore (2002). Lastly, participants reported demographic information such as age and gender.

### Results

#### Manipulation Checks^10^

Two coders, who were unaware of the design and research hypothesis, independently coded the descriptions provided by the participants into two categories. Descriptions focusing on the image as a whole were coded as “1,” descriptions referring to a specific area of the image were coded as “2.” The pre-discussion consensus between the coders was 88%. Disagreements were discussed until consensus was reached. 96.2% of the descriptions in the global condition focused on the overall image (vs. 11.7% in the local condition), whereas 88.3% of the descriptions in the local condition focused on a specific area of the image (vs. 3.8% in the global condition). In the control condition, 46.2% of the descriptions focused on the overall image, and 53.8% of the descriptions focused on a specific area of the image.

#### Effect of Processing on Positive Mood

As our hypothesis focused on positive mood, and as some research has demonstrated the independence of positive and negative affect from each other (Warr et al., 1983; Diener and Emmons, 1984; Cacioppo and Berntson, 1994; Barrett and Russell, 1998), we analyzed positive and negative mood separately in the following reports.

We averaged all the positive mood items to yield an aggregated index of positive mood (Cronbach α = 0.82) and conducted an ANCOVA with this index as the dependent variable, the experimental condition as the independent variable, and the pre-manipulation positive mood as the covariate. We found a significant main effect of condition, *F*(2, 66) = 3.80, *p* = 0.027, partial η^2^ = 0.10^11^. Supporting our prediction, when controlling for pre-manipulation positive mood, participants in the global processing condition (adjusted marginal *M* = 4.81, *SE* = 0.13) reported higher positive mood than those in both the local processing condition (adjusted marginal *M* = 4.34, *SE* = 0.15), *p* = 0.025, 95% CI [0.06, 0.86], *d* = 0.730, and those in the control condition (adjusted marginal *M* = 4.38, *SE* = 0.13), *p* = 0.020, *d* = 0.730, 95% CI [0.07, 0.79], *d* = 0.730. Participants in the latter two groups did not differ from each other, *p* = 0.874, 95% CI [−0.43, 0.37].

#### Effects of Processing on Negative Mood

We averaged the negative mood items to yield an aggregated index of negative mood (Cronbach α = 0.87) and conducted an ANCOVA with pre-manipulation negative mood as a covariate. There was no significant main effect of condition on participants’ negative mood, *F*(2, 66) = 0.16, *p* = 0.86, partial η^2^ = 0.005. Thus, processing styles affected participants’ positive mood, but not negative mood (adjusted marginal *M* = 2.53, *SE* = 0.20 for the local condition; adjusted marginal *M* = 2.52, *SE* = 0.16 for the global condition; adjusted marginal *M* = 2.64, *SE* = 0.16 for the control condition).

## Meta-Analysis

Many of the studies in the current paper may be considered as underpowered in today’s standard because of the relatively small sample sizes, although it was still a common practice when we first conducted the research in 2008. To address this limitation, we integrated the evidence from these four studies and conducted a meta-analysis to increase the statistical power of the test for the overall effect of perceptual processing on positive mood (see Cohn and Becker, 2003, for review; Hunter and Schmidt, 1990, p. 75).

### Computation of Effect Sizes

In all four studies, participants engaged in either a global or local perceptual processing task, and reported their positive mood afterward. We decided that the standardized mean difference, *d*, defined as the difference between the means of the two groups (global vs. local) divided by a pooled estimate of the standard deviations for both groups, best represented the effect-size index for each of these studies. Therefore, positive effect sizes indicated a more positive mood rating after global than local processing, whereas negative effect sizes indicated more positive mood after local than global processing.

### Analyses of Effect Sizes

**FIGURE 2:**
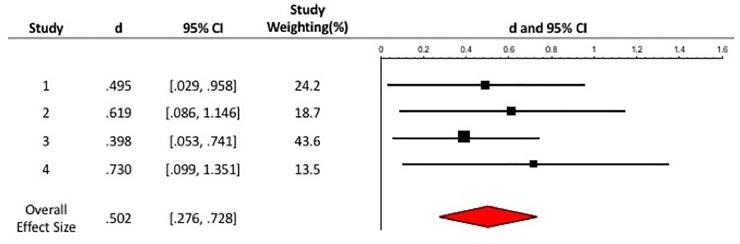
Forest plot of a meta-analysis based on a random-effects model on the effects of global cognitive processing style on positive mood, and the respective effect sizes (*d*), 95% Confidence Intervals (CI), and weights for each study.

The relevant statistics of the effect size estimates are presented together with a forest plot (see Figure 2). All four studies were included in this meta-analysis, involving a total of 308 participants. Each of these studies was weighted according to the sample size of the study as well as the variance of their respective effect sizes. Hence, studies with larger sample size and smaller variance were given more weight. The distribution of effect sizes was homogeneous, *Q*(3) = 1.008, *p* = 0.799. Assuming a random-effects model, the overall effect size, *d* = 0.502, 95% CI [0.28, 0.73] was significant, *z* = 4.358, *p* < 0.001. Thus, the effect of global perceptual processing in improving one’s positive mood, compared to local perceptual processing, was evident, supporting our hypothesis.

## Discussion

Past research indicates that the global perspective often takes precedence over the local perspective (Navon, 1977). The reason for such a global advantage, according to previous research, may be sensory, or attentional (Kimchi, 1992). Our findings suggest that global processing may also have an affective advantage over local processing.

In four studies, using different global vs. local processing manipulations (i.e., a Navon letters, landscape pictures, and Google Maps pictures), we found that global processing led people to be happier (Studies 1–3) or in a more positive mood (Study 4) than local processing did. This finding was further supported by a meta-analysis of all four studies. This is the first evidence, to our knowledge, that has demonstrated the effect of global vs. local perceptual processing on mood. More specifically, people reported being happier or in a more positive mood after engaging in global processing than local processing. As we did not find any evidence for the effect of perceptual processing on people’s negative mood, we will focus our discussion on the effects of global vs. local processing on people’s positive mood.

Does global processing increase people’s positive mood, or does local processing decrease people’s positive mood? The evidence is inconclusive. In Studies 1–3, where sad mood was induced first, the simple effects showed that global processing sustained (but not necessarily enhanced) participants’ happy mood and local processing decreased their happy mood. In Study 4, where sad mood was not induced at first, local processing did not seem to decrease people’s positive mood, but global processing increased their positive mood, compared to the control condition. Thus, it is possible that global processing can enhance and local processing can dampen positive mood, depending on the specific context. Nonetheless, the relative advantage of global processing over local processing in sustaining or elevating positive mood was consistent across all four studies.

Why does global processing lead to more positive mood than local processing? One concept that is closely related to the broadening of mental horizons or a global perceptual scope is the idea of abstraction (for a review, see Burgoon et al., 2013). Updegraff and Suh (2007) found that people who construed their lives more abstractly evaluated themselves more positively and reported higher life satisfaction than people who construed their lives more concretely. We believe that focusing on the global picture activates a “big picture” schema causing observers to adopt a global perspective. This global perspective could possibly lead to a relative increase in happiness because, compared to local processing, global processing may nudge the observer to evaluate the information around them or their lives more abstractly, thereby potentially trivializing more minor and immediate concerns. This global mindset could also elicit more experiences of positive emotions as it could selectively broaden our attention to the positive stimuli around us, thereby increasing the probability of finding positive meaning in life (Fredrickson, 2000). Nonetheless, these are just speculations. It will be important for future research to examine the mechanism underlying the effect of perceptual processing on mood.

The causal effect of perceptual processing on mood is also consistent with the literature on depression. Cognitive distortions are considered as a maintenance mechanism perpetuating low mood associated with depression (Beck, 2008). One of these cognitive distortions may be a bias to focus on local rather than global processing. In fact, de Fockert and Cooper (2014) found that depression is associated with a decreased tendency to prioritize global-level processing. Participants with low levels of depression showed global processing bias and responded faster to global stimuli than local ones (Navon stimuli), but participants with high levels of depression did not exhibit such a global-processing bias. Consistent with mood maintenance motivations (Isen, 1987; Wegener et al., 1995; Clore et al., 2001), people with lower levels of depression may be more motivated to engage in global instead of local processing in order to maintain their relative positive mood or to avoid mood reduction (Bless and Fiedler, 1995; Bless et al., 1996; Gasper and Clore, 2002). Assuming people with depression are motivated to elevate their mood, they may not be relying on an effective strategy. For example, they may be engaging in local more than global processing. Compared to global and schematic processing, local processing is more detail oriented and likely involves more effort, and thus engaging in local processing may draw more of people’s attention to specifics that could potentially reduce one’s positive mood (Clore et al., 2001). The present findings on the causal link between global (vs. local) level of processing and positive mood suggest that it might be helpful to target processing biases when treating depression. Of course, stronger evidence is needed in order to make informative recommendations for treatment.

The present research is in line with some of the past research that argues for a reciprocal relationship between thinking and mood. Just as positive and negative moods can lead to different processing styles, different processing styles can also influence mood. From a cognitive neuroscience perspective, Bar (2009) proposes a reciprocal relationship between breadth of associative thinking and mood, such that positive mood broadens one’s associative thinking and attentional scope, and broader associative thinking in turn promotes better mood. They also found evidence that affective value and associate processing share a cortical substrate (Shenhav et al., 2013). In parallel to this research, our findings suggest that the relationship between mood and perceptual processing styles may also be reciprocal and self-perpetuating, such that happy mood promotes global processing, and global processing leads to happier moods relative to local processing. This is not to say that positive mood and global perceptual processing is always favorable or always a good thing. When mood is the focus, and the sole goal is to feel happy, this reciprocation between positive mood and global processing may be beneficial because it helps to maintain one’s positive mood. However, when feeling happy is not the goal (or not the only goal) and systematic processing associated with problem solving is valued instead, local processing may be more adaptive, despite the affective disadvantage.

### Limitations and Future Studies

Future research should address some of the limitations in the present research. First, the initial negative mood induction in Studies 1–3 could invite an alternative interpretation of our findings. That is, instead of a direct effect of global processing promoting positive mood, it is possible that global processing led to a more positive mood than local processing because the broadened global processing could have made it easier for participants to stop ruminating on the sad movie that participants watched at the beginning of the study. On the other hand, narrowed local processing could have made it harder for participants to stop ruminating on the sad movie, thus prolonging their negative mood state. Although this is possible, it does not explain all the findings in the paper, as Study 4 did reveal a consistent finding without the initial mood manipulation. Furthermore, if this alternative explanation were true, then a reverse pattern of effect would be expected with an initial happy mood induction. An unpublished study that we ran in our lab, however, did not show such an opposite pattern of results when we induced positive mood initially^12^. Nonetheless, future research should further examine this possibility.

Second, the studies reported here relied on relatively small sample sizes (although similar sample sizes were common when these studies were conducted years ago). This limitation in power makes the current research more exploratory. Future research should attempt to replicate the effect with better powered studies. Furthermore, our research was based on a group of white, educated, and relatively rich university students in an industrialized society (“WEIRD” samples, Henrich et al., 2010), who tend to be happy and feel positive in general. Future research may examine whether these findings can be generalized to other populations.

One may argue that the yellow or red box used to delimit a local target area of the images in the local condition could affect participants’ mood in some ways, thus acting as a potential confound. We think this is possible but unlikely. In a separate pretest, we showed participants either the images as a whole, or the local images delineated by the boxes (with boxes shown), and found no differences in the ratings of pleasantness. Therefore, it seems unlikely that the colored boxes would have caused the observed effects.

Although we provided some evidence for a causal effect of perceptual processing on mood, the mechanism underlying this effect remains unclear. Some evidence in the current study suggests that people’s happiness decreased after local processing, instead of increasing after global processing. Future studies should investigate the mechanisms directly and explore potential boundary conditions. It is unclear how robust the effect may be outside the lab and how durable it is, which should be examined in future research as well.

## Conclusion

In conclusion, we have demonstrated in a set of studies that, relative to local processing, global processing is more conducive to positive mood. Together with previous research showing the effect of mood on cognitive processing, the present research suggests that the relationship between mood and processing style can be causally bidirectional and self-perpetuating (at least for positive mood), which can have far-reaching implications for mood regulation in general.

## Ethics Statement

This study was carried out in accordance with the recommendations of the Tri-Council Guidelines (Article 6.14) and Standard Operating Procedures (405.001), General Research Ethics Board at Queen’s University with written informed consent from all subjects. All subjects gave written informed consent in accordance with the Declaration of Helsinki. The protocol was approved by the General Research Ethics Board at Queen’s University.

## Author Contributions

Project conception and idea was contributed by L-JJ. Methodology and design of the study was contributed by L-JJ, SY, MB, and KM. Data collection was done by SY, KM, and MB. Data analyses and writing was done by SY and L-JJ (MB and KM also contributed).

## Conflict of Interest Statement

The authors declare that the research was conducted in the absence of any commercial or financial relationships that could be construed as a potential conflict of interest.

## Footnotes

1 As there were no prior studies or work for us to estimate the effect size, we aimed to recruit 30–40 participants per condition, in combination with a timeline (i.e., end of the term).

2 Indeed, six participants reported a happiness rating of nine or ten out of a 10-point scale even after watching the sad movie. This implied that they were either not paying attention to the movie or were not serious in reporting their mood. Thus, these participants were excluded from the analyses. Pattern of results remained the same before and after excluding these participants.

3 Without controlling for pre-manipulation happy mood, the condition main effect was significant, *F*(1, 72) = 9.20, *p* = 0.003, partial η^2^ = 0.11. We also performed a Bayesian ANCOVA in JASP (Morey and Rouder, 2011; Rouder and Morey, 2012) with JZS prior and default prior scale (Cauchy prior scale parameter for covariates = 0.354). There is strong evidence for the alternative hypothesis than the null hypothesis of no processing effect, BayesFactor > 150 (BF10 = 1.925 × 1020). The condition and pre-manipulation mood model was preferred to the pre-manipulation mood only model (BF10 = 1.305 × 1020) by a factor of 1.48 (1.925/1.305 = 1.475), which is considered as weak evidence for our prediction that that processing has an effect on post-manipulation happiness, after controlling for pre-manipulation happiness.

4 There was no significant difference between conditions in participants’ performance accuracy (99.32% vs. 99.33%), *t*(78) = 0.123, *p* = 0.902, or in reaction time, *t*(78) = 1.142, *p* = 0.257. Thus, participants in both global and local conditions were equally fast and accurate in their responses.

5 As in Study 1, four participants reported a happiness rating of nine or ten out of the 10-point happiness mood scale even after watching the sad movie, and thus were excluded from the analyses. Pattern of results remained the same before and after excluding these participants.

6 Without controlling for pre-manipulation happy mood, the condition main effect was marginally significant, *F*(1, 56) = 3.38, *p* = 0.071, partial η^2^ = 0.06. A Bayesian ANCOVA in JASP revealed a strong evidence for the alternative hypothesis than for the null hypothesis of no processing effect, BayesFactor > 150 (BF10 = 5.949 × 108). The condition and pre-manipulation mood model was preferred to the pre-manipulation mood only model (BF10 = 2.508 × 108) by a factor of 2.38 (5.949/2.508 = 2.372), which is considered as weak evidence in support of our prediction that processing has an effect on post-manipulation happiness, after controlling for pre-manipulation happiness.

7 One participant reported a happiness rating of nine out of the 10-point happiness mood scale after watching the sad movie, and thus was excluded from the analyses. Pattern of results remained the same before and after excluding this participant.

8 Without controlling for pre-manipulation happy mood, the condition main effect was not significant, *F*(1, 131) = 2.03, *p* = 0.156, although the pattern of results (i.e., participants in the global condition were happier than those in the local condition) remained consistent with the other studies. A Bayesian ANCOVA in JASP revealed strong evidence for the alternative hypothesis than for the null hypothesis of no processing effect, BayesFactor > 150 (BF10 = 7.97 × 1017). The condition and pre-manipulation mood model was preferred to the pre-manipulation mood only model (BF10 = 4.013 × 1017) by a factor of 1.99 (7.970/4.013 = 1.986), which is considered weak evidence in support of our hypothesis that processing affected people’s post-manipulation happiness after controlling for their pre-manipulation mood.

9 Following a similar procedure from Förster et al. (2008), we created the control condition in this way (half global and half local stimuli), acknowledging the difficulty of coming up with a neutral task of similar nature that does not involve either local or global processing.

10 We acknowledge that the lack of manipulation checks in study 2 and 3 was a limitation, and attempted to address this limitation by including a manipulation check in this study.

11 Without controlling for pre-manipulation happy mood, the condition main effect was marginally significant, *F*(2, 67) = 2.87, *p* = 0.063. A Bayesian ANCOVA in JASP revealed strong evidence for the alternative hypothesis than for the null hypothesis of no processing effect, BayesFactor > 150 (BF10 = 5.735 × 107). The condition and pre-manipulation mood model was preferred to the pre-manipulation mood only model (BF10 = 2.663 × 107) by a factor of 2.15 (5.735/2.663 = 2.153), which is considered weak evidence in support for our hypothesis that processing affected participants’ post-manipulation positive mood, after controlling for their pre-manipulation mood.

12 We followed a procedure similar to Studies 1 and 3 (i.e., participants’ global and local processing were manipulated using Navon’s letter task and Google image task, respectively, in two separate studies), but induced an initial happy mood by showing participants a happy movie at the beginning of the study. Participants in the local condition did not report a happier mood than those in the global condition.
